# Tendencies towards emigration and their association with introversion and ethnocentrism among final-year medical students from Osijek, Croatia: a cross-sectional study

**DOI:** 10.1186/s12909-023-04611-8

**Published:** 2023-09-03

**Authors:** Jakov Milić, Zrinka Vuksan-Ćusa, Jelena Jakab, Maja Ćurčić, Livia Puljak, Iva Milić Vranješ, Maja Miškulin

**Affiliations:** 1 Croatian Carmelite Province of St. Joseph, Zagreb, Croatia; 2 School of Medicine, University of Zagreb, Zagreb, Croatia; 3 Faculty of Medicine Osijek, Josip Juraj Strossmayer University of Osijek, Osijek, Croatia; 4 Faculty of Dental Medicine and Health Osijek, Josip Juraj Strossmayer University of Osijek, Osijek, Croatia; 5 University Hospital Dubrava, Zagreb, Croatia; 6 Center for Evidence-Based Medicine and Health Care, Catholic University of Croatia, Ilica 242, Zagreb, 10000 Croatia

**Keywords:** Emigration, Medical students, Ethnocentrism, Introversion, Medical specialty

## Abstract

**Background:**

The migration of healthcare workers is attracting growing attention worldwide. Attitudes towards emigration develop over the years, and it is possible that, in addition to social factors, they are influenced by the characteristics of a person’s personality and the sense of belonging to the social environment. This study aimed to determine the tendencies of final-year medical students’ from Osijek, Croatia, towards emigration after graduation and after specialization, as well as their specialty preferences and to investigate whether introversion and ethnocentrism have an impact on attitudes toward leaving the country in search of employment elsewhere.

**Methods:**

A cross-sectional study was conducted among final-year (6th year) medical students from Osijek, Croatia, in two consecutive academic years – 2014/2015 and 2015/2016. Students completed a questionnaire about sociodemographic characteristics, academic and scientific performance, preferences about their future medical career, the medical specialty of choice, emigration tendencies after graduation and specialization, ethnocentrism and introversion.

**Results:**

There were 124 students who participated in the study (response rate: 96%). A quarter of participants agreed that they are likely or very likely to emigrate after graduation (25.0%) or after specialization (23.39%). Variables associated with the intention to emigrate were specialty preferences (students that prefer endocrinology and psychiatry had the highest emigration tendencies), academic year in which the participants were included (students included in 2014/2015 were more prone to emigrate after specialization, p = 0.060), prior involvement in scientific projects (students with experience in scientific projects expressed higher tendencies to emigrate after graduation, p = 0.023), and ethnocentrism (higher ethnocentrism was associated with a lower tendency towards emigration after specialization, Spearman’s rho = -0.191).

**Conclusion:**

Our finding that a quarter of final-year medical students from the Faculty of Medicine Osijek were considering emigrating from Croatia in search of employment elsewhere after graduation or specialization is not as high as in previous Croatian studies or studies conducted in other European countries. Even though these data may be encouraging, considering the lack of physicians in Croatia, interventions are needed to prevent permanent emigration to protect the future functioning of the Croatian health system. Furthermore, our study did not find significant associations between levels of introversion and ethnocentrism and tendency to emigrate from Croatia. It seems that the phenomenon covering the emigration of students is more complex and influenced by many other factors which were not included in our study.

**Supplementary Information:**

The online version contains supplementary material available at 10.1186/s12909-023-04611-8.

## Background

The migration of healthcare workers is attracting growing attention worldwide [1]. Trained healthcare professionals, which are in demand worldwide, migrate looking for better standards of living and quality of life, financially more rewarding jobs, access to advanced technology and more stable political conditions. People tend to migrate from developing to developed countries, which can devastate health systems in developing countries [2]. The World Health Organization (WHO) recognizes such migration as a cause of the uneven distribution of the global health workforce, which may lead to even greater inequalities in healthcare availability [3].

Recent trends in the international migration of physicians show that the United States of America (USA) is their main destination country, followed by the United Kingdom and Germany [4]. Since Croatia joined the European Union (EU) in June 2013, many medical professionals have emigrated from Croatia to more developed countries, with more physicians emigrating each year. Most Croatian physicians migrated to Germany, Slovenia, the United Kingdom (UK), Ireland and Switzerland [5]. Studies have shown that about 140 physicians leave Croatia annually [6], making Croatia one of the three EU countries from which most physicians emigrate [5].

Furthermore, a study among Croatian students from 2014 indicated that as many as 35% of students wanted to emigrate after Croatia joined the EU, expressing readiness to work and live abroad permanently. Most of the surveyed students wanted to move to Germany (40%), the USA and Canada (17%), and the UK (11%) [7].

In a study conducted among medical students in Croatia, the most common reasons for wanting emigration were higher earnings, secure work, better health system organization, more opportunities for career advancement, and greater respect for medical personnel in society [8].

One study has shown that healthcare workers who reported a tendency to emigrate were mostly men, young, not married, and more educated [9], while the other revealed a correlation between the tendency to permanently work out of their country and being abroad before for a study purpose or having a relative or friend abroad [10]. In addition, another study found a negative correlation between interest in leaving their county for work and wanting to pursue a surgical career [11].

Attitudes towards emigration develop over the years, and it is possible that, in addition to social factors, they are influenced by the characteristics of a person’s personality and the sense of belonging to the social environment. Ethnocentrism can be used as a measure of a feeling of belonging to the social environment. The term ethnocentrism was first introduced by Sumner in 1906, defining it as an understanding of the world in which an individual puts their group at the center of everything while evaluating all other groups in relation to it [12].

Ethnocentrism is defined within the framework of two terms: “ingroup” - a social group in which an individual feels like a member of it, and “outgroup” - the rest of the social environment with which the individual cannot identify [13]. Ethnocentric behavior is based on specific ties within the group, such as language, accent, physical features, and religion, and has a highly developed territorial component [14]. Since, to the authors’ knowledge, this concept was not previously analyzed in the context of migration, and since it intuitively seems to lead in the direction of a lower tendency to migrate, we decided to analyze the impact of ethnocentrism on the tendency to migrate.

Another potential variable that can play a role in the decision on the choice of future profession, as well as the place of residence, is the introversion of the respondent. The first concept of introversion and extraversion was introduced by Jung in 1921, characterizing them as two main personality types [15]. He described introversion as an orientation towards inner experience and a tendency towards introspective cognitive activity. In later conceptualization attempts, introverts were described as persons preoccupied with inner ideas and feelings [16] and prone to social withdrawal [17]. Eysenck describes introverts as calm, serious, passive and pessimistic people, who have few close friends, plan ahead, are not irritable and value ethical principles [18]. Previous research has shown that extraversion is positively associated with the tendency to migrate since extraverted persons have an easier time making interpersonal connections in a new environment [19].

Since brain drain can profoundly affect the region, we also aimed to assess academic success by self-reported grade point average (GPA), previous involvement in scientific research, whether the students failed a year of study and its relation to the tendency to emigrate. Previous research showed no association between academic success and the tendency to migrate [8].

Since involvement in scientific research often implies international cooperation and having contacts in other countries [20], we hypothesized that the students who participated in scientific research would have a higher tendency for emigration.

Studies regarding emigration tendencies are highly relevant for Croatia because of its very high emigration flows. Emigration has particularly intensified after Croatia joined the EU. Analysis of immigration data from the national statistical offices of the core EU countries indicated that 230 thousand people left Croatia between 2013 and 2016 [21]. Eastern Croatia, whose largest city is Osijek, is an economically less developed region in Croatia and one of the Croatian regions with a ratio of migrants to a domestic population close to or above 1% [21]. Thus, this region warrants further emigration-related studies.

This study aimed to determine the tendencies of final-year medical students’ from Osijek, Croatia, towards emigration after graduation and after specialization, as well as their specialty preferences and to investigate whether introversion and ethnocentrism have an impact on attitudes toward permanently leaving the country in search of employment elsewhere after graduation and after specialization.

## Methods

### Study design

This was a cross-sectional study.

### Participants

The study included two generations of final-year (6th year) medical students from the Faculty of Medicine Osijek. The study was conducted during two consecutive academic years, 2014/2015 and 2015/2016, and each was conducted during the last months of the academic year, May and June 2015 and 2016.

### Ethics

The study was approved by the Ethics Committee of the Faculty of Medicine Osijek, and all participants gave written informed consent. All methods were carried out in accordance with relevant guidelines and regulations.

#### Questionnaire

The students were given a self-administered anonymous paper-based questionnaire consisting of two parts. Full text of the questionnaire can be found in Supplementary file 1. The first part included eight questions about the participants’ sociodemographic characteristics: age, sex, year of study, self-reported GPA (in Croatia, the GPAs are on a scale 1.00–5.00, where grade 1 is a fail, and grades 2–5 are passing grades), whether they failed a year of study, whether they participated in scientific research, preferences about their future career in medicine and their medical specialty of choice.

The second part of the questionnaire included questions about emigration tendencies after graduation and specialization (questions 9 and 10) and validated scales for determining levels of ethnocentrism (questions 11–32) and introversion (questions 33–50). Questions about emigration tendencies were formulated as positive sentences (“I am very likely to emigrate after graduation.” and “I am very likely to emigrate after specialization.”) after which the participants rated their level of agreement to statements on a 5-point Likert scale as follows: “Strongly Disagree” “Disagree”, “Neither Disagree nor Agree”, “Agree” and “Strongly Agree”.

McCroskey introversion scale by McCroskey and Richmond was used to determine the participants’ personality types [22]. On 18 items, participants rated their level of agreement to statements on a 5-point Likert scale, where “Strongly Disagree” “Disagree”, “Neither Disagree nor Agree”, “Agree” and “Strongly Agree” represent the options that were coded 1 through 5, respectively. Questionnaire results were calculated by the accumulation score’s formula that should be in the range of 12 to 60. On that account, scores 12–24 represent highly extraverted; scores 25–35 are categorized as moderate extraversion, 37–48 as moderate introversion and 49–60 are classified as high introversion. Example items from the scale are „Do you like to have many social engagements?“ and „Do you usually take the initiative in making new friends?“.

Ethnocentrism was assessed using the Revised Ethnocentrism Scale [23]. Respondents needed to note the degree to which they agree or disagree with each item using the following five­point scale: Strongly Disagree = 1; Disagree = 1; Neutral = 3; Agree = 4; Strongly Agree = 5. Questions 15, 18 and 20 were reverse-scored, and questions 14, 17, 23, 26, 27, 28 and 30 were dropped, as suggested by the author of the original instrument [23]. Based on the 15 measurement items, the score range was 15 to 75, with a mid-point of 45. The higher the score is, the higher the ethnocentrism is. Example items from the scale are „Most other cultures are backward compared to my culture.“ and „Most people would be happier if they lived like people in my culture.“.

### Statistical analysis

The distribution normality of numerical variables was tested by Kolmogorov-Smirnov and Shapiro-Wilks test. Non-parametrical tests were used due to the non-normal distribution of all scalar variables. Numerical data were presented as medians and interquartile ranges (IQR), or as means and standard deviations (SD). Categoric variables were presented by relative and absolute frequencies. Mann-Whitney U-test was used to assess the differences between the two groups of students based on the academic year in which the participants were included (2014/2015 and 2015/2016). Chi-square test was used to assess the difference among ratios between independent samples, whereas Spearman’s ρ was used to assess correlations among variables. Data analysis was performed using an IBM SPSS Statistics version 16.0 for Windows. P-values less than 0.05 were considered statistically significant.

### Raw data

Raw data collected within the study are provided in Supplementary file 2.

## Results

A total of 124 final-year medical students were included in the study, of which 34 (27%) were men. In the 2014/2015 academic year, 60 students were enrolled for the final year and in the 2015/2016 academic year, 69 students were enrolled for the final year, so the response rate was 96%. Of the total number of participants, 61 (49%) were tested in the 2014/2015 academic year, and 63 (51%) in the 2015/2016 academic year. The mean age of the participants was 24.14 ± 1.074 (range 23–30).

Most students indicated they would prefer a career in clinical medicine (94%) (Table 1). The most popular specializations were Pediatrics and Internal medicine (11% each), followed by Otorhinolaryngology, Surgery, and Anesthesiology (7% each). The mean GPA was 4.13 (0.379).

**Table 1:** Academic characteristics of included students (N = 124)

		n (%)
Failed a year		
	Yes	24 (19)
	No	100 (81)
Participated in a scientific project		
	Yes	39 (31)
	No	85 (69)
Area of medical interest		
	Basic medical sciences	2 (2)
	Clinical medicine	117 (94)
	Public health	5 (4)

The tendency toward emigration after graduation was expressed by 31 (25%) students, while 29 (24%) of them indicated they would emigrate after specialization (Table 2). In our sample, students that prefer endocrinology and psychiatry as their specialty had the highest median scores on the questions pertaining to emigration tendency (Table 3).

**Table 2:** Tendencies towards emigration of final-year medical students and the differences between the two tested generations (N = 124)

		n (%)			
		All students	2014/2015 academic year	2015/2016 academic year	Chi Square (p value)
I am very likely to emigrate after graduation.					
	Strongly Disagree	24 (19)	7 (11)	17 (27)	8.028 (0.091)
	Disagree	27 (22)	14 (23)	13 (21)	
	Neutral	42 (34)	23 (38)	19 (30)	
	Agree	19 (15)	8 (13)	11 (17)	
	Strongly Agree	12 (10)	9 (15)	3 (5)	
I am very likely to emigrate after specialization.					
	Strongly Disagree	18 (14)	5 (8)	13 (20)	13.238 (0.010)
	Disagree	33 (27)	16 (26)	17 (27)	
	Neutral	44 (35)	19 (31)	25 (40)	
	Agree	17 (14)	10 (17)	7 (11)	
	Strongly Agree	12 (10)	11 (18)	1 (2)	

**Table 3:** Specialty preferences and median scores of the questions pertaining to emigration

	n	(%)	Median (IQR)	
Specialty			Emigration after graduation	Emigration after specialization
Anesthesiology	9	(7.4)	3 (1.5–4.5)	3 (1.5-4)
Cardiology	5	(4.1)	3 (1.5-4)	2 (1.5-4)
Cardiothoracic Surgery	3	(2.5)	3 (2)	3 (2)
Child and Adolescent Psychiatry and Psychotherapy	1	(0.8)	3	3
Clinical Genetics	1	(0.8)	5	5
Dermatology and Venereology	2	(1.6)	2.5 (2)	2.5 (2)
Endocrinology	2	(1.6)	4 (3)	4 (3)
Gastroenterology	4	(3.3)	2.5 (1.25–4.5)	2.5 (1.25–4.5)
Gynecology and Obstetrics	3	(2.5)	2 (1)	2 (1)
Infectious Diseases	2	(1.6)	3 (2)	3 (2)
Internal Medicine	14	(11.5)	3 (1.75-4)	3 (2-4.25)
Medical Microbiology	1	(0.8)	3	4
Nephrology	3	(2.5)	3 (1)	3 (2)
Neurology	7	(5.7)	3 (2–4)	3 (2–3)
Neurosurgery	1	(0.8)	3	3
Occupational Medicine	3	(2.5)	3 (1)	3 (1)
Ophthalmology	6	(4.9)	3 (2.75–4.25)	3 (2.75–4.25)
Oro-Maxillo-Facial Surgery	2	(1.6)	1.5 (1)	2 (2)
Orthopedics	3	(2.5)	2 (1)	2 (1)
Otorhinolaryngology	9	(7.4)	2 (1–3)	2 (1–3)
Pediatrics	14	(11.5)	2 (1–4)	3 (1–3)
Pathology	1	(0.8)	1	1
Physical Medicine and Rehabilitation	2	(1.6)	2.5 (2)	2.5 (2)
Plastic, Reconstructive and Aesthetic Surgery	1	(0.8)	3	3
Pneumology	1	(0.8)	3	3
Psychiatry	5	(4.1)	4 (2-4.5)	4 (2.5–4.5)
Public Health Medicine	4	(3.3)	3 (1.5–3.75)	3.5 (2.25-4)
Radiology	4	(3.3)	2.25 (3-4.5)	2.25 (3-4.5)
Surgery	9	(7.4)	3 (2-4.5)	3 (1.5-3)

Acronym: IQR = interquartile range

The mean score (SD) for ethnocentrism was 32.58 (6.129), and the median score (IQR) of the introversion scale was 41 (36–44). Higher ethnocentrism was associated with a lower tendency towards emigration after specialization (Table 4).

**Table 4:** Correlations between the GPA, Introversion and Ethnocentrism scores and the inclination towards emigration after graduation (Graduation) and after specialization (Specialization) (N = 124)

	Graduation	Specialization	Introversion	Ethnocentrism
GPA	0.007	-0.037	0.004	0.070
Graduation		0.686^**^	0.108	-0.135
Specialization			0.061	-0.191^*^
Introversion				0.161

*p < 0.05, Spearman’s rho

**p < 0.01, Spearman’s rho

No differences were observed in the tendency to emigrate after graduation (Mann Whitney test, z=-0.029, p = 0.977) or specialization (Mann Whitney test, z=-0.305, p = 0.760) between students who did and did not fail a study year.

Significant differences between generations were observed regarding the tendencies to emigrate after specialization (Mann Whitney test, z=-2.773, p = 0.006); students enrolled for the final study year in the 2014/2015 academic year were more prone to emigrate. Medians and IQRs of the two groups were 3 [2–4] vs. 3 [2, 3], respectively. No differences were observed in the tendency to emigrate after graduation (Mann Whitney test, z=-1.882, p = 0.060) between the two generations.

No sex-related differences in the tendency to emigrate were observed, either after graduation (Mann Whitney test, z=-1.165, p = 0.244), or after specialization (Mann Whitney test, z=-0.607, p = 0.544).

Students that participated in scientific projects during their medical education expressed a higher tendency towards emigrating after graduation than students who did not participate in scientific projects (Mann Whitney test, z=-2.268, p = 0.023), medians and IQRs 3 [2–4] and 3 (1.5-3), respectively. No differences were observed in the tendency to emigrate after specialization regarding participation in scientific projects (Mann Whitney test, z=-1.244, p = 0.213).

## Discussion

In our study, a quarter of final-year medical students from the Faculty of Medicine Osijek, Croatia, agreed that they are very likely to emigrate after graduation or after specialization. Variables associated with the tendency to emigrate were prior involvement in scientific projects, GPA and ethnocentrism, and differences in the median tendency to migrate were observed between students of different specialty preference.

The percentage of students who considered leaving the country in search of employment abroad in our study differs from a study conducted in 2013, which included final-year medical students from all four medical schools in Croatia (in Zagreb, Rijeka, Split, and Osijek). Among 260 participants included in the 2013 study, 90 (35%) indicated the tendency to emigrate [7]. Moreover, the percentage of students from our study who expressed a wish to seek employment abroad is not as high as in other European countries. For example, 62.1% of Polish students indicated having a plan to seek employment abroad after graduation [1].

Regarding the standards of care, it is noteworthy that in Croatia, these standards are generally lower compared to EU averages. Croatia had 344 physicians per 100 000 inhabitants in 2018. Since the EU average is 382 physicians per 100 000 inhabitants, Croatian numbers are below the EU average. Also, the number of general practitioners per 100 000 inhabitants (57 in 2019) in Croatia was below the EU average (78 in 2013) and physicians in Croatia are needed particularly in primary care [24].

Given that Croatia already lacks physicians, with the number of physicians below the EU average, the percentage of students with a tendency to emigrate should be perceived as worrisome. This is particularly alarming considering the projection of the Croatian Medical Chamber stating that Croatia will lose a third of its physicians due to emigration and retirement by 2025 [6]as well as increasing needs for healthcare services due to extended life expectancy. Thus, raising awareness of the importance of attracting healthcare workers to stay in the homeland is crucial.

Furthermore, our findings suggest that students who participated in scientific projects during their medical education had a stronger tendency to emigrate after graduation. We could not compare these results with others since we did not find such data in the literature. We can speculate that participation in scientific projects during medical education is associated with higher motivation and ambition for collaborating with foreign colleagues to improve scientific accomplishments.

We found no significant association between levels of introversion and tendency to emigrate. To the best of our knowledge, this is the first study that investigated the link between introversion and tendency to emigrate after graduation or specialization among medical students. However, we found a study that investigated the association between personality traits and migration intentions among university students in Germany [19]. Our findings are not in accordance with that study since they found a positive association between migration and extraversion, implying a negative correlation between introversion and migration intentions [19].

Likewise, a significant association between levels of ethnocentrism and a tendency toward emigration was not observed. To our best knowledge, this is also the first study to investigate the association between ethnocentrism and the tendency to emigrate.

In the larger context, it has to be mentioned that the migration of medical personnel also derives some benefits to countries they leave behind, particularly in the case of temporary migration. In more advanced settings, physicians can acquire new skills, gain more experience and improve their research skills.

At the same time, retaining enough physicians for healthcare systems to function is very important. This is why the WHO Global Code of Practice on the International Recruitment of Health Personnel in May 2010 called for more ethical hiring of health workers to evade active recruitment in countries that already cope with an insufficient number of health care professionals [25].

Furthermore, countries that have invested in the education of young health professionals experience a loss of financial resources when these people leave the country since highly educated personnel are one of the most expensive resources in any country. However, these countries are not at a loss only because of the investment in the health workers, but also due to the possible contribution of those workers to the health system [2]. For instance, the total cost of educating one physician from primary school to university in Kenya is US$ 65,997; and for every physician who emigrates, a country suffers a loss of about US$ 517,931 worth of investment [26]. We were not able to find in the literature any comparable studies that were conducted in Croatia.

It is important to highlight that our study was conducted at a very specific time point in the geopolitical EU history – shortly after Croatia joined the EU in June 2013. Thus, our data provide insight into the emigration tendencies of medical students in a region that is economically disadvantaged and highly affected by population outflow, early after EU accession [21]. As such, our data will serve as a benchmark for future studies that can compare future data with ours.

Also, it should be highlighted that ethnocentrism is a concept rarely analyzed. While, this was limiting in the sense that making comparisons with other research was difficult, this is also an advantage of our study, as our findings contribute to a research field that was insufficiently explored.

### Limitations of the study

It could be considered that with 124 students included, our study had a relatively small sample size. However, we had a response rate of 96%, thus adequately representing the population of medical students in Eastern Croatia. The Faculty of Medicine Osijek is the only Medical School in Eastern Croatia. For this region, the results about the tendencies for emigration are crucial, as Eastern Croatia is one of the areas of Croatia that is experiencing the highest levels of emigration.

Second, we compared two generations of students that are only one year apart from each other. This minimal age difference makes generational comparisons challenging to interpret. We included two consecutive generations to increase the sample size because the school in which we conducted the study is fairly small and enrolls a limited number of students per year. However, despite the minimal age differences, we also wanted to compare the two generations and see if the minimal age difference affects the tendency to migrate, hypothesizing that the younger generation would be more prone to migration. It is known that young people make up a part of the population that is most likely to emigrate [27]. Since we found significant differences between the two consecutive generations, we believe that these results could be interesting to the readers, and useful for fostering further research. We were unable to find comparable studies in the literature to compare our results with those from other authors, but our study could motivate other researchers to explore how quickly intergenerational tendencies towards emigration change and why. We have added this now to the manuscript.

Third, the student population was predominantly women, with only 27% men participants. However, this should not be considered a limitation of the study because this is representative of the trend of so-called feminization of medicine, as the majority of students enrolled and graduated from medical studies in Croatia are women [28]. Moreover, other variables should have been taken into account, such as Big-5, marital status, financial status, fluency in foreign languages, or previous student mobility.

The survey was conducted in 2015 and 2016. However, the results are relevant for the number of reasons. Firstly, the data presented are novel as we were unable to find a single other study that has explored ethnocentrism among medical students in Croatia. Furthermore, we were unable to find other studies in the literature that have investigated the link between introversion and tendency to emigrate after graduation or specialization among medical students. Also, we were unable to find a single other study in the literature that has investigated the association between ethnocentrism and the tendency to emigrate. Secondly, it has been posited that all research should be published [29]. Research is the advancement of knowledge and ideally, all research findings should be publicly available. Knowledge gained via research is a common good. Publication of research results is one of the prerequisites for verifying results and improving and increasing knowledge [29]. Thirdly, the topic that we have explored is an important topic for individual countries and health systems. It has been highlighted in the literature that the migration of medical professionals as a result of the expansion of the European Union is cause for concern, but that there is a significant lack of information available about this phenomenon [30]. Our study fills part of this gap. Fourthly, our results will serve as a benchmark to show the status of the tendency to emigrate shortly after joining the EU. Newer research can take these results as a point of comparison, especially after Croatia joined the Schengen Area in January 2023 and after the widespread protests of physicians related to their wages and work conditions that were organized in March 2023 in Croatia.

## Conclusion

This study showed that almost one-fourth of final-year medical students from the Faculty of Medicine Osijek were considering emigrating from Croatia in search of employment elsewhere after graduation or specialization. These results are not as high as they were in previous Croatian studies or studies conducted in other European countries. Even though these data may be encouraging, considering the lack of physicians in Croatia, interventions are needed to prevent permanent emigration to protect the future functioning of the Croatian health system.

Furthermore, our study did not find significant associations between levels of introversion and tendency to emigrate from Croatia, but we did find a significant negative association between the levels of ethnocentrism and willingness to migrate after specialization. It seems that the phenomenon covering the emigration of students is more complex and influenced by many other factors which were not included in our study.

Further studies are needed to explore more factors associated with the migration tendencies of medical personnel. In future studies, it would be interesting to investigate what other personality traits (internal factors), such as other dimensions of the Big-5, marital and financial status, or patriotism, influence the decision to emigrate after graduation or specialization, as well as external factors such as public policies and strategies.

### Acronyms

EU European Union.

GPA Grade point average.

IQR Interquartile ranges.

SD Standard deviation.

UK United Kingdom.

USA United States of America.

WHO World Health Organization.

### Electronic supplementary material

Below is the link to the electronic supplementary material.

Supplementary Material 1

Supplementary Material 2

## Data Availability

Raw data collected and analyzed within this study are publicly available in Supplementary file 2.

## Abbreviations

EU: European Union
GPA: Grade point average
IQR: Interquartile ranges
SD: Standard deviation
UK: United Kingdom
USA: United States of America
WHO: World Health Organization
