# Supplementary material for: Tendencies towards emigration and their association with introversion and ethnocentrism among final-year medical students from Osijek, Croatia: a cross-sectional study
Source: BMC Med Educ. 2023 Sep 3;23:632. doi: 10.1186/s12909-023-04611-8 (PMC10476414; doi:10.1186/s12909-023-04611-8)
Supplement: Supplementary file 1 — Supplementary Material 1 [file 12909_2023_4611_MOESM1_ESM.docx]

**Supplementary file 1. The questionnaire used in the study**

**We kindly ask you to answer the following questions (please circle or write your answer):**

1. Gender: MALE FEMALE

2. Age: ______

3. School year: 1 2 3 4 5 6

4. Grade point average: ______

5. Did you fail at least a year in medical school? YES NO

6. Were you involved in any scientific projects during your studies? YES NO

7. Pertaining to your future career in medicine, what would you prefer?

a) Basic medical sciences (e.g. anatomy, physiology, histology)

b) Clinical medicine

c) Public health

d) Other. State your preference: ___________________

8. Please specify your specialty of choice. Pick ONE of the following:

1. Allergology
2. Anaesthesiology
3. Cardiology
4. Cardiothoracic Surgery
5. Child and Adolescent Psychiatry and Psychotherapy
6. Clinical Genetics
7. Clinical Neurophysiology
8. Dermatology and Venereology
9. Emergency Medicine
10. Endocrinology
11. Gastroenterology
12. Geriatrics
13. Gynaecology and Obstetrics
14. Infectious Diseases
15. Internal Medicine
16. Laboratory Medicine / Medical Biopathology
17. Medical Microbiology
18. Nephrology
19. Neurology
20. Neurosurgery
21. Nuclear Medicine
22. Occupational Medicine
23. Ophthalmology
24. Oro-Maxillo-Facial Surgery
25. Orthopaedics
26. Otorhinolaryngology
27. Paediatric Surgery
28. Paediatrics
29. Pathology
30. Physical Medicine and Rehabilitation
31. Plastic, Reconstructive and Aesthetic Surgery
32. Pneumology
33. Psychiatry
34. Public Health Medicine
35. Radiology
36. Radiotherapy
37. Rheumatology
38. Surgery
39. Thoracic Surgery
40. Urology
41. Vascular Surgery

Please indicate the degree to which you agree or disagree with each item using the following five-point scale:

Strongly Disagree = 1; Disagree = 1; Neutral = 3; Agree = 4; Strongly Agree = 5;

|  | I am very likely to emigrate after graduation. | 1 | 2 | 3 | 4 | 5 |
| --- | --- | --- | --- | --- | --- | --- |
|  | I am very likely to emigrate after specialization. | 1 | 2 | 3 | 4 | 5 |
|  | Most other cultures are backward compared to my culture. | 1 | 2 | 3 | 4 | 5 |
|  | My culture should be the role model for other cultures. | 1 | 2 | 3 | 4 | 5 |
|  | People from other cultures act strange when they come to my culture. | 1 | 2 | 3 | 4 | 5 |
|  | Lifestyles in other cultures are just as valid as those in my culture. | 1 | 2 | 3 | 4 | 5 |
|  | Other cultures should try to be more like my culture. | 1 | 2 | 3 | 4 | 5 |
|  | I am not interested in the values and customs of other cultures. | 1 | 2 | 3 | 4 | 5 |
|  | People in my culture could learn a lot from people in other cultures. | 1 | 2 | 3 | 4 | 5 |
|  | Most people from other cultures just don't know what's good for them. | 1 | 2 | 3 | 4 | 5 |
|  | I respect the values and customs of other cultures. | 1 | 2 | 3 | 4 | 5 |
|  | Other cultures are smart to look up to our culture. | 1 | 2 | 3 | 4 | 5 |
|  | Most people would be happier if they lived like people in my culture. | 1 | 2 | 3 | 4 | 5 |
|  | I have many friends from different cultures. | 1 | 2 | 3 | 4 | 5 |
|  | People in my culture have just about the best lifestyles of anywhere. | 1 | 2 | 3 | 4 | 5 |
|  | Lifestyles in other cultures are not as valid as those in my culture. | 1 | 2 | 3 | 4 | 5 |
|  | I am very interested in the values and customs of other cultures. | 1 | 2 | 3 | 4 | 5 |
|  | I apply my values when judging people who are different. | 1 | 2 | 3 | 4 | 5 |
|  | I see people who are similar to me as virtuous. | 1 | 2 | 3 | 4 | 5 |
|  | I do not cooperate with people who are different. | 1 | 2 | 3 | 4 | 5 |
|  | Most people in my culture just don't know what is good for them. | 1 | 2 | 3 | 4 | 5 |
|  | I do not trust people who are different. | 1 | 2 | 3 | 4 | 5 |
|  | I dislike interacting with people from different cultures. | 1 | 2 | 3 | 4 | 5 |
|  | I have little respect for the values and customs of other cultures. | 1 | 2 | 3 | 4 | 5 |
|  | Are you inclined to keep in the background on social occasions? | 1 | 2 | 3 | 4 | 5 |
|  | Do you like to mix socially with people? | 1 | 2 | 3 | 4 | 5 |
|  | Do you sometimes feel happy, sometimes depressed, without any apparent reason? | 1 | 2 | 3 | 4 | 5 |
|  | Are you inclined to limit your acquaintances to a select few? | 1 | 2 | 3 | 4 | 5 |
|  | Do you like to have many social engagements? | 1 | 2 | 3 | 4 | 5 |
|  | Do you have frequent ups and downs in mood, either with or without apparent cause? | 1 | 2 | 3 | 4 | 5 |
|  | Would you rate yourself as a happy-go-lucky individual? | 1 | 2 | 3 | 4 | 5 |
|  | Can you usually let yourself go and have a good time at a party? | 1 | 2 | 3 | 4 | 5 |
|  | Are you inclined to be moody? | 1 | 2 | 3 | 4 | 5 |
|  | Would you be very unhappy if you were prevented from making numerous social contacts? | 1 | 2 | 3 | 4 | 5 |
|  | Do you usually take the initiative in making new friends? | 1 | 2 | 3 | 4 | 5 |
|  | Does your mind often wander while you are trying to concentrate? | 1 | 2 | 3 | 4 | 5 |
|  | Do you like to play pranks upon others? | 1 | 2 | 3 | 4 | 5 |
|  | Are you usually a "good mixer?" | 1 | 2 | 3 | 4 | 5 |
|  | Are you sometimes bubbling over with energy and sometimes very sluggish? | 1 | 2 | 3 | 4 | 5 |
|  | Do you often "have the time of your life" at social affairs? | 1 | 2 | 3 | 4 | 5 |
|  | Are you frequently "lost in thought" even when you should be taking part in a conversation? | 1 | 2 | 3 | 4 | 5 |
|  | Do you derive more satisfaction from social activities than from anything else? | 1 | 2 | 3 | 4 | 5 |

**We thank you for your participation!**
